# Granular Cell Tumor Mimicking Breast Carcinoma: A Report of Two Cases

**DOI:** 10.7759/cureus.57500

**Published:** 2024-04-03

**Authors:** Masahiro Ito, Masakazu Amari, Akiko Sato, Masahiro Hikichi

**Affiliations:** 1 Breast Surgery, Tohoku Kosai Hospital, Sendai, JPN; 2 Breast Surgery, Tohoku Kosai Hosital, Sendai, JPN

**Keywords:** myoblastoma, contrast-enhanced magnetic resonance imaging, case report, breast cancer, granular cell tumor

## Abstract

Granular cell tumor (GCT) of the breast is a rare neoplasm that can mimic the clinical and radiological features of breast carcinoma. This paper presents two case reports - a rare male case and a more common female case - to underline the diagnostic challenges posed by GCT in the breast. The male patient was initially suspected of having a breast tumor based on mammography and ultrasound findings. The female patient also exhibited radiological signs suggestive of breast cancer. In both cases, the mammograms showed irregular lesions, while ultrasounds revealed solid masses with posterior shadowing and echogenic halos, mimicking carcinoma. Dynamic contrast-enhanced magnetic resonance imaging (MRI) suggested benign patterns in both cases, but only histopathologic examination post-core needle biopsy confirmed the diagnosis of GCT. These cases highlight the variability of GCT imaging presentations and the potential for misdiagnosis as breast carcinoma. The tumors exhibited distinct histopathological features, such as large polygonal cells with granular eosinophilic cytoplasm and S100 protein, differentiating them from breast carcinoma. However, imaging alone proved insufficient for diagnosis, emphasizing the need for histopathologic confirmation. The report discusses the importance of including GCT in differential diagnoses and utilizing core needle biopsy for accurate evaluation. Both cases had no recurrence during follow-up after wide resection, indicating a favorable prognosis for GCT when properly managed.

## Introduction

Granular cell tumor (GCT) of the breast is rare, with a reported frequency of 0.1% and 0.7% of all breast tumors [1-3]. Although typically benign neoplasms, they are known for their potential to mimic the clinical and radiological features of breast carcinoma. Initially described by Abrikossoff in 1926, GCT is thought to arise from Schwann cells and can occur in a variety of anatomic sites, including the breast, most commonly the skin, followed by the head and neck, especially the tongue [4,5]. GCT is most often reported in women but can also occur in the male breast, comprising about 10% [6,7]. The diagnostic challenge with GCT lies in its ability to mimic more common and aggressive forms of breast cancer, both in imaging and clinical examination. These tumors often present as firm, painless, and irregular masses, similar to findings in breast carcinoma. Radiologically, GCT can show features such as spiculated margins or nonparallel orientation, which is typically associated with malignant breast tumors. Due to these similarities, GCT can often be misdiagnosed as breast carcinoma, leading to unnecessary or more aggressive treatments. Sonographically guided biopsy of the lesion is the diagnostic procedure of choice [6]. Histologically, GCTs are characterized by sheets or clusters of large, round to polygonal cells with eosinophilic granular cytoplasm and are positive for staining with S100 and CD68 [6]. Here, we present two cases of GCT mimicking breast carcinoma. One case was male, which is rare among GCTs, and the other was female. The purpose of the report is to recognize GCT as one of the differential diagnoses and to provide appropriate treatment.

## Case presentation

Patient 1

**Figure 1:**
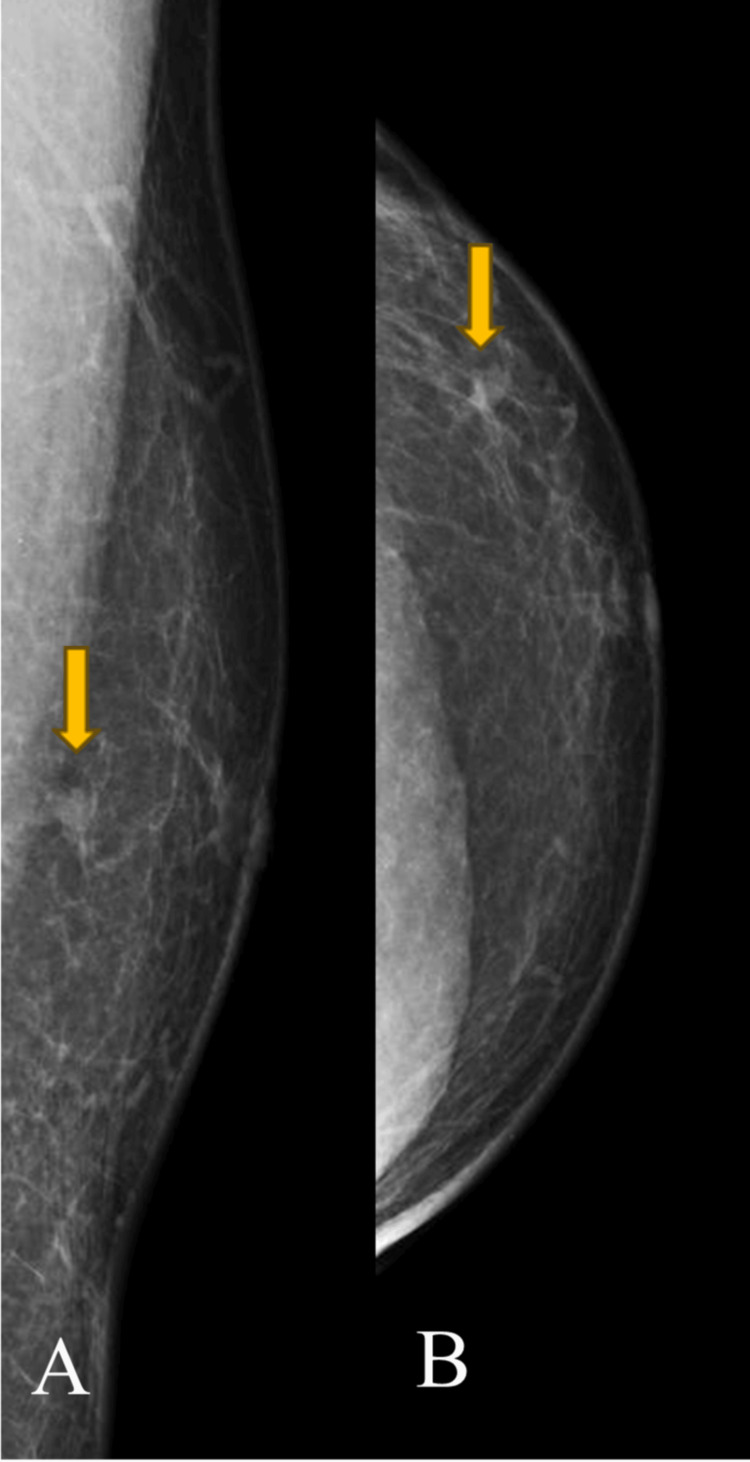
(A) Left mediolateral oblique and (B) left craniocaudal mammograms showing focal asymmetry in the inferior lateral quadrant.

**Figure 2:**
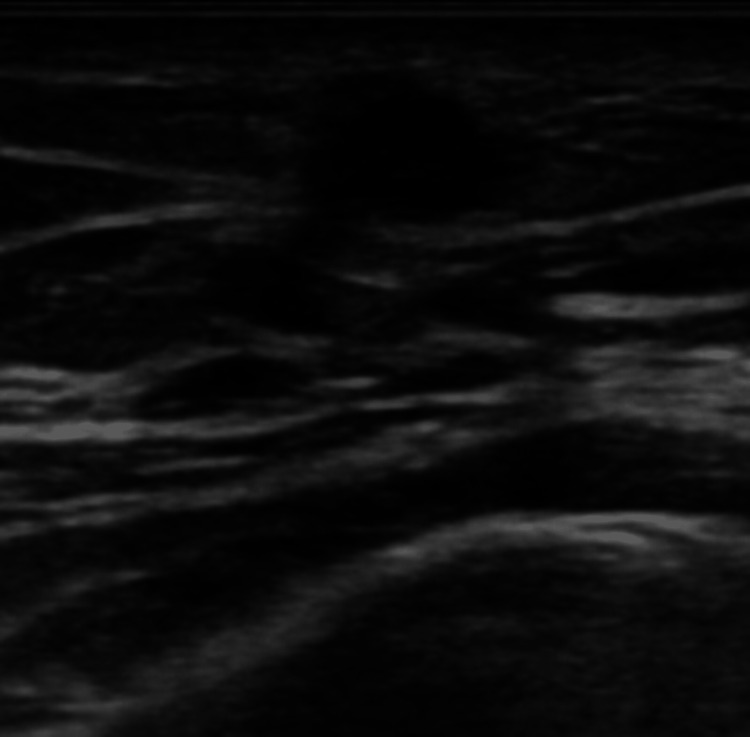
Breast ultrasonography shows an irregular hypoechoic, microlobulated mass parallel to the skin surface, with areas of ill-defined margins and no definite posterior acoustic enhancement or shadowing.

**Figure 3:**
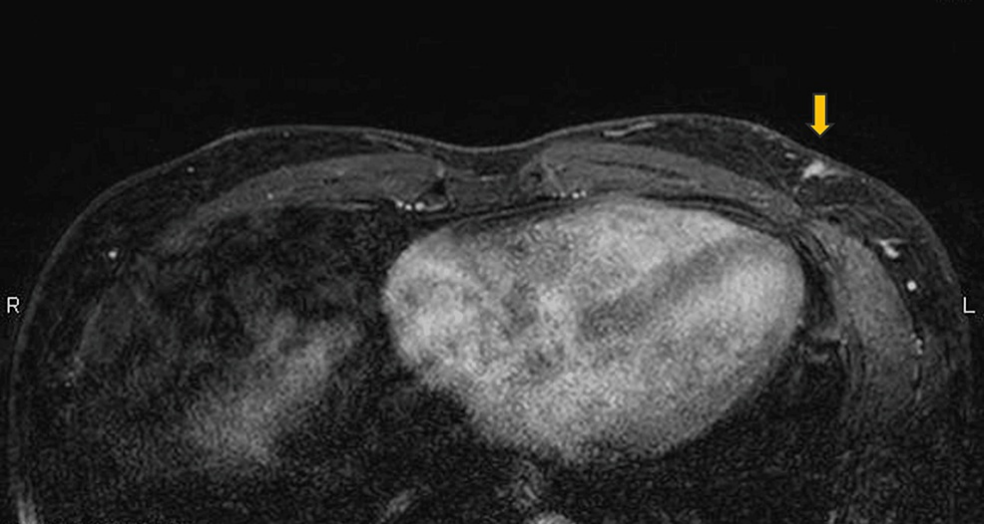
Dynamic contrast-enhanced MRI showing a 9 mm mass with mild progressive enhancement pattern (type I curves). MRI, magnetic resonance imaging

**Figure 4:**
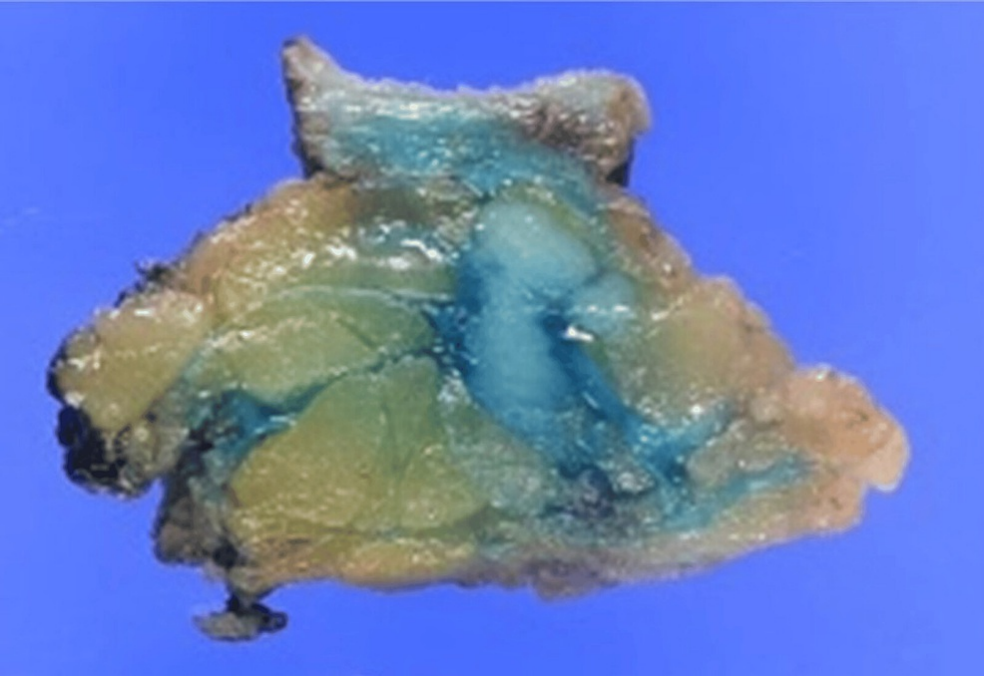
Gross appearance of the cross-sectional surface of the resected specimen.

**Figure 5:**
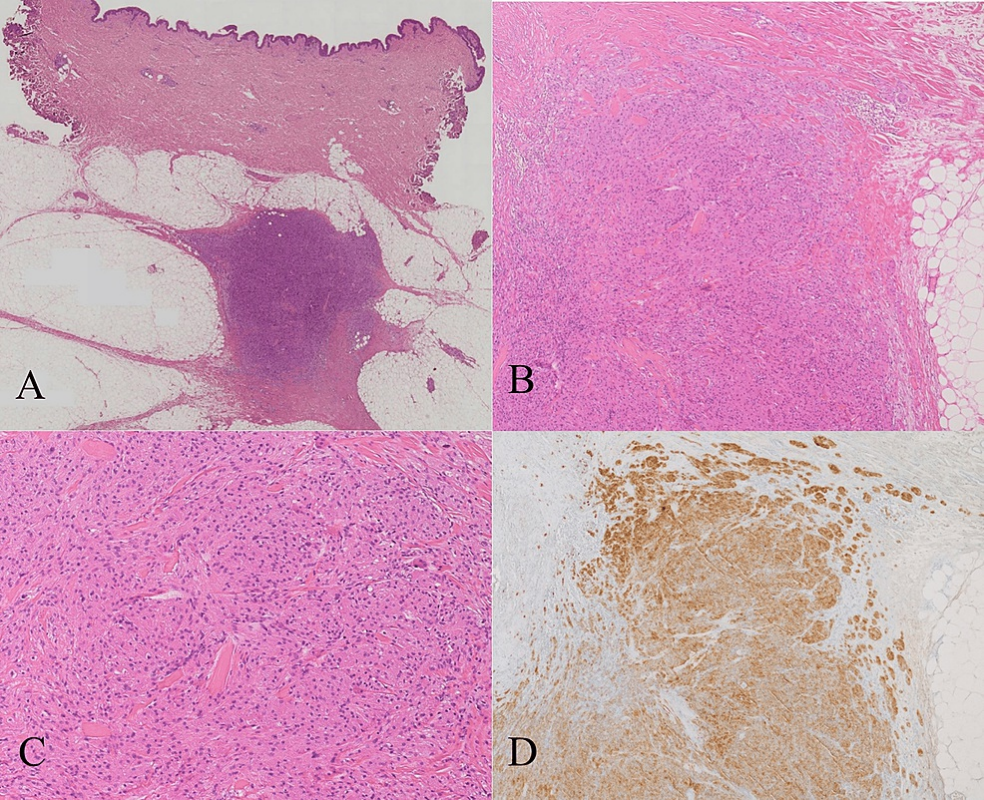
(A) The Loupe image showing a 7 mm mass with irregular margins but relatively well-defined borders; the tumor mildly extends along the septal wall of adipose tissue and around blood vessels; (B) low magnification (H&E stain, ×40) showing a moderately cellular tumor composed of infiltrating nests of polygonal cells; (C) high magnification (H&E stain, ×100) showing tumor cells with small, oval, bland-appearing nuclei and abundant granular eosinophilic cytoplasm; and (D) immunohistochemical staining for S100 showing diffuse positivity in tumor cells at ×40. H&E, hematoxylin and eosin

A 62-year-old male presented with a lump in the lower outer quadrant of his breast. The patient's family history was significant, with three relatives having a history of breast cancer. Clinical examination revealed a 1.2 cm × 1.0 cm mass on the four o'clock position of the left breast. Mammography findings were categorized as Breast Imaging Reporting and Data System (BI-RADS) 3 (Figure 1). Breast ultrasonography identified an irregular 5 mm × 4 mm × 5 mm mass categorized as BI-RADS 4b at the same site, indicating a possibility of breast cancer (Figure 2). Dynamic contrast-enhanced magnetic resonance imaging (MRI) found that the mass is subcutaneous, a feature that has been recognized as a clue to the diagnosis of GCT (Figure 3). A core needle biopsy was performed for definitive diagnostic purposes and revealed the presence of GCT. The patient then underwent a wide local excision of the mass (Figure 4). Histopathological examination confirmed the diagnosis of a benign GCT (Figure 5). It has been 42 months since the surgery, and no local recurrence has been observed. 

 

Patient 2

**Figure 6:**
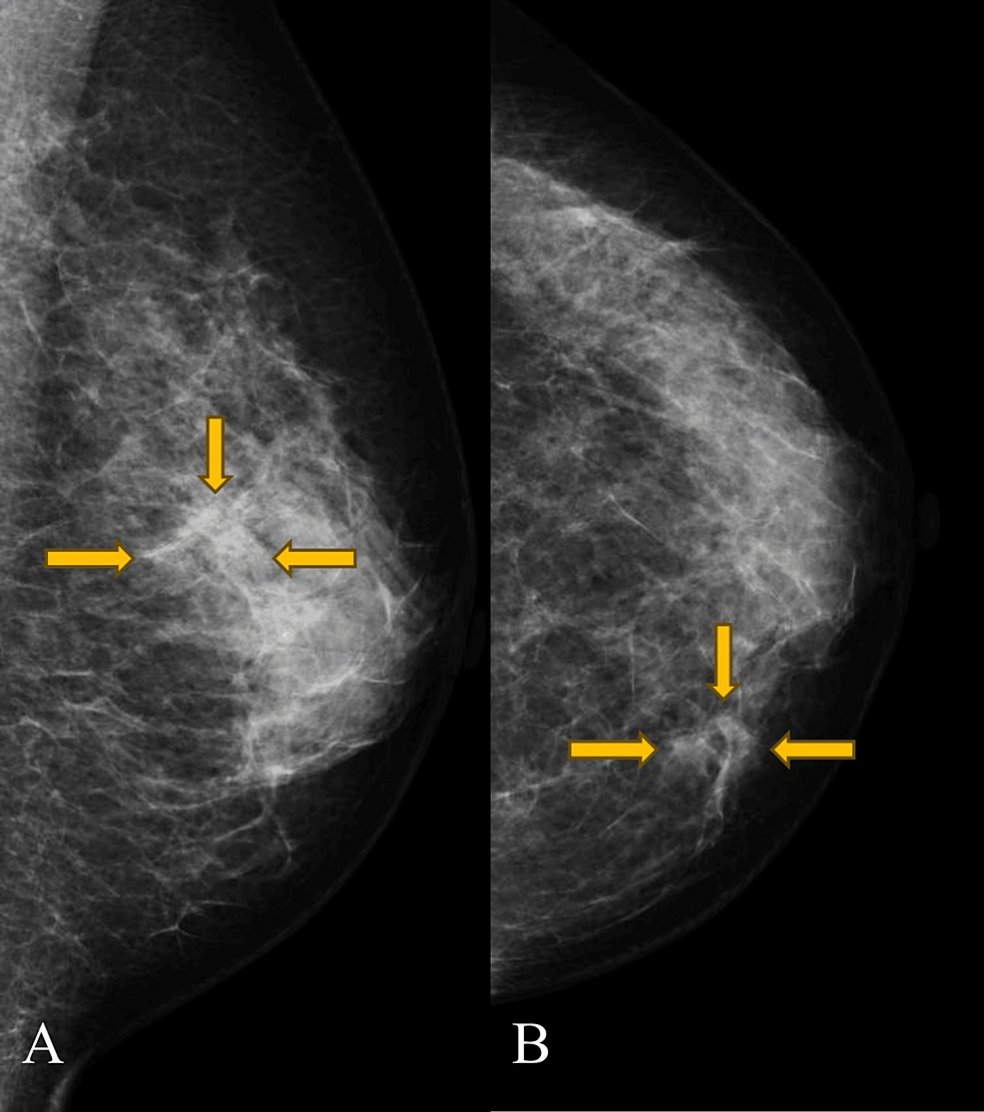
(A) Left mediolateral oblique and (B) left craniocaudal mammograms showing architectural distortion in the superior medial quadrant.

**Figure 7:**
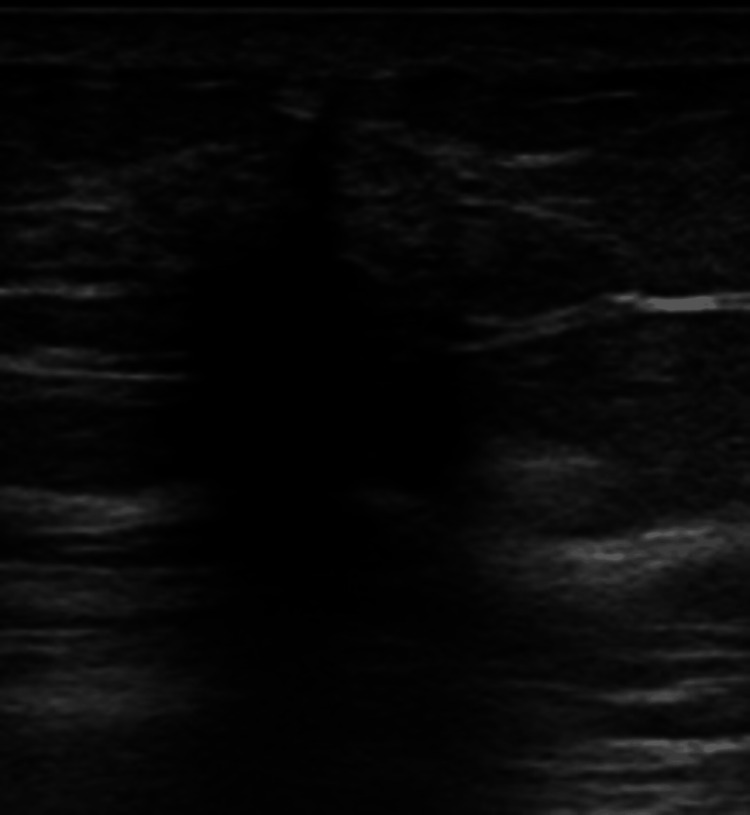
Breast ultrasound showing an irregular hypoechoic mass with indistinct margins and posterior acoustic shadowing.

**Figure 8:**
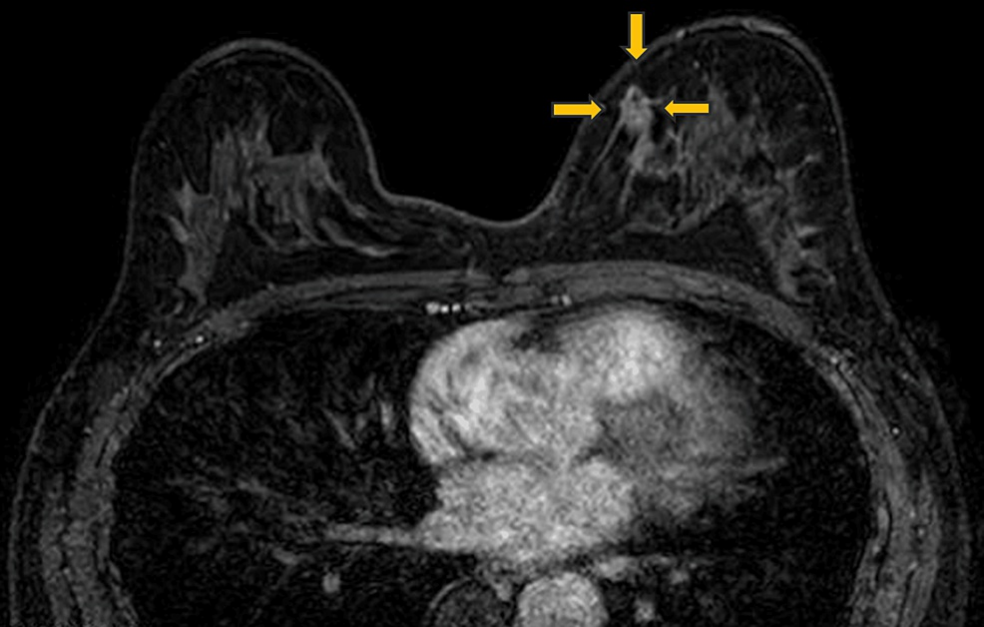
Dynamic contrast-enhanced MRI image showing a 10 mm mass with a slow-persistent enhancement pattern (type I curves). MRI, magnetic resonance imaging

**Figure 9:**
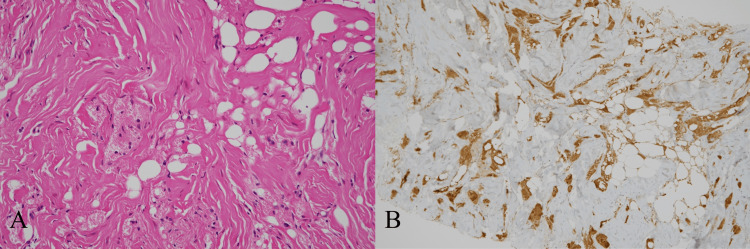
(A) High magnification (H&E stain, ×200) showing tumor cells with abundant granular eosinophilic cytoplasm; (B) immunohistochemical staining for S100 showing diffuse positivity in tumor cells at ×100. H&E, hematoxylin and eosin

**Figure 10:**
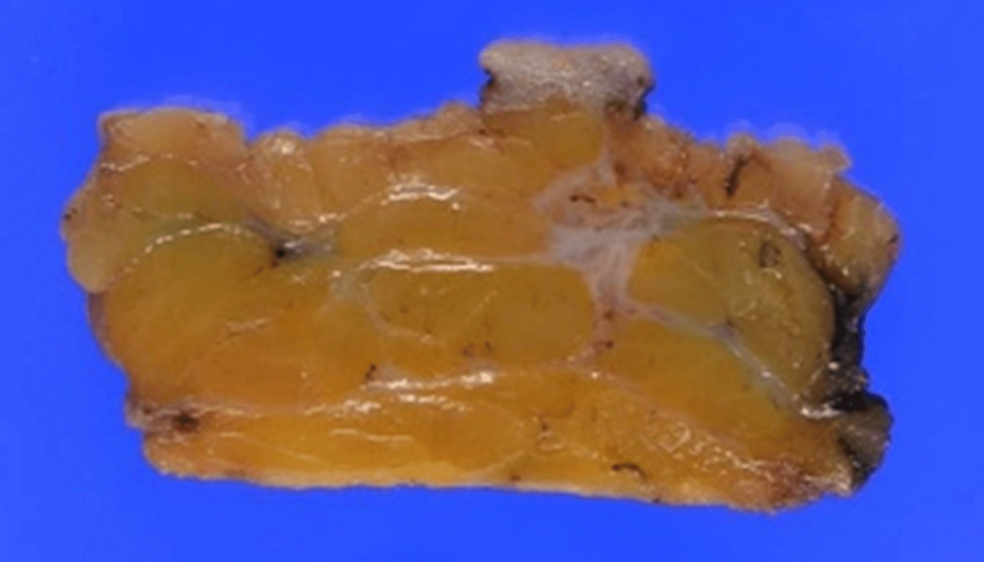
Gross appearance of the cross-sectional surface of the resected specimen.

**Figure 11:**
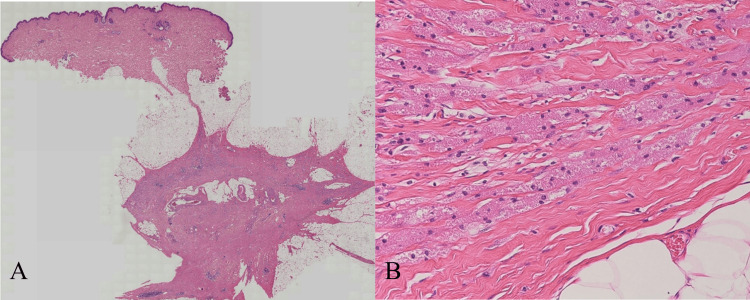
(A) The Loupe image showing a 13 mm mass growing in the interstitial portion of the mammary tissue and in the septal portion of the adipose tissue, forming a nodular lesion with indistinct borders; (B) high magnification (H&E stain, ×200) showing infiltrating nests of tumor cells with coarsely granular cytoplasm. H&E, hematoxylin and eosin

A 50-year-old woman, who had undergone a right lobe thyroidectomy seven years earlier for papillary thyroid cancer, was referred to our hospital with a left breast mass as her primary concern. On examination, a 1 cm mass was palpated in the upper inner quadrant of the left breast. Mammography findings were classified as BI-RADS 4 (Figure 6), and subsequent breast ultrasonography revealed an irregular hypoechoic tumor measuring 8 mm × 7 mm × 5 mm in the same area, raising suspicion of breast cancer (Figure 7). Dynamic contrast-enhanced breast MRI showed a 10 mm mass with a slightly higher signal than the mammary gland, and the time-intensity curve (TIC) showed a benign-like pattern (Figure 8). Based on these findings, a core needle biopsy was performed, and GCT was suspected (Figures 9A, 9B). The patient subsequently underwent a wide local excision of the tumor (Figure 10). Histopathological examination of the excised tissue confirmed the diagnosis of a benign GCT (Figures 11A, 11B). It has been 16 months since the surgery, and there has been no local recurrence.

## Discussion

GCT is an intriguing entity in the spectrum of breast neoplasms. The frequency of GCT in the breast is approximately 5%, constituting a very small percentage of breast tumors [8]. This rarity can often lead to a lack of familiarity among clinicians and radiologists, contributing to potential misdiagnosis. In 1946, Haagensen and Stout described five patients and emphasized the importance of distinguishing this benign tumor from carcinoma [9]. The clinical presentation of GCT usually presents with a firm or hard, painless mass. GCT may arise in any part of the breast parenchyma, including the axillary tail, or in a subcutaneous location. In terms of imaging, GCT on mammography typically shows ill-defined or spiculated lesions, which is similar to malignant breast carcinoma [10]. Ultrasound findings often reveal a solid mass with posterior shadowing, suggesting carcinoma. Additionally, GCT may have a strong echogenic halo, a feature that can be noted in the adjacent parenchyma [11]. MRI findings in GCT of the breast are also nonspecific, and the available image reports are limited [12]. In both of our two cases, breast ultrasound showed imaging findings suspicious for breast cancer, but MRI showed a benign tumor pattern. Therefore, it is difficult to differentiate GCT from breast cancer based on imaging alone, emphasizing the need for histopathologic confirmation. Histologically, GCT is characterized by large polygonal cells with granular eosinophilic cytoplasm and is positive for S100 protein and CD68. Despite these distinct histopathological features, the initial resemblance to malignancies on imaging can mislead clinicians. Thus, accurate diagnosis of GCT relies heavily on the combination of imaging and careful pathological examination. The distinction between GCT and breast carcinoma, especially apocrine carcinoma, is particularly difficult in fine needle aspiration cytology specimens [13]. A core needle biopsy is necessary for the diagnosis of GCT. In both cases presented here, breast cancer was suspected on imaging diagnosis, but a core needle biopsy was able to distinguish them from breast cancer. It is estimated that 1% to 2% of GCTs are malignant. Wide excision is recommended in the treatment of GCT [14]. The infiltrative nature of the tumor often leads to positive margins after initial excision. Incomplete excision can cause local recurrence. No recurrence was observed after follow-up in our cases.

## Conclusions

This case report emphasizes the diagnostic challenges presented by GCT in the breast, particularly when they mimic breast carcinoma in clinical and radiological presentations. Our cases highlight the necessity of including GCT in the differential diagnosis of breast masses, especially given their potential to resemble more common and aggressive forms of breast cancer. In conclusion, our report contributes to the understanding of GCT in the breast, underlining the importance of considering these rare tumors in differential diagnoses and using core needle biopsy for accurate histopathological evaluation. This approach is vital to ensure appropriate patient management and avoid the potential consequences of misdiagnosis.
